# Increased Efficacy of Brentuximab Vedotin (SGN-35) in Combination with Cytokine-Induced Killer Cells in Lymphoma

**DOI:** 10.3390/ijms17071056

**Published:** 2016-07-01

**Authors:** Laura Esser, Hans Weiher, Ingo Schmidt-Wolf

**Affiliations:** 1 Center for Integrated Oncology (CIO), Medizinische Klinik und Poliklinik III, University of Bonn, Sigmund-Freud-Straße 25, 53105 Bonn, Germany; laura92esser@googlemail.com; 2 Hochschule Bonn-Rhein-Sieg, 53359 Rheinbach, Germany; Hans.Weiher@h-brs.de

**Keywords:** lymphoma, cytokine-induced killer (CIK) cells, antibody–drug conjugate, SGN-35, CD30^+^ cells

## Abstract

Brentuximab vedotin (SGN-35) is an antibody–drug conjugate with a high selectivity against CD30^+^ cell lines and more than 300-fold less activity against antigen-negative cells. In the last years, the results of many in vitro and in vivo studies have led to the fast approval of this drug to treat lymphoma patients. Another innovative method to treat tumor cells including lymphoma cells is the use cytokine-induced killer (CIK) cells, which have also been approved and proven to be a safe treatment with only minor adverse events. In this study, a possible additive effect when combining SGN-35 with CIK cells was investigated. The combinational treatment showed that it reduces the viability of CD30^+^ cell lines significantly in vitro. Additionally, the amount of lymphoma cells was significantly reduced when exposed to CIK cells as well as when exposed to SGN-35. A significant negative effect of SGN-35 on the function of CIK cells could be excluded. These results lead to the assumption that SGN-35 and CIK cells in combination might achieve better results in an in vitro setting compared to the single use of SGN-35 and CIK cells. Further investigations in in vivo models must be conducted to obtain a better understanding of the exact mechanisms of both treatments when applied in combination.

## 1. Introduction

Tumor cells of Hodgkin’s disease (HD) and anaplastic large cell lymphomas (ALCLs) express the tumor necrosis factor receptor (TNFR) family member CD30, making it a diagnostic marker for those lymphomas. Healthy tissues outside the immune system or resting monocytes and lymphocytes, on the other hand, do not express the surface antigen CD30. Thus, due to the restricted expression of CD30, it serves as an ideal target for antibody-directed therapies (mAb therapy) [1,2]. In recent years, there have been approaches with monoclonal antibodies directed against lymphoma cells showing promising results. In most cases, monoclonal antibodies are directed against lymphocyte surface antigens [3,4,5,6]. Brentuximab vedotin (SGN-35) is one of those antibody–drug conjugates shown to have antitumor activity in vitro and in vivo against HD and ALCL. It is a chimeric anti-CD30 monoclonal antibody (cAC 10) that is combined via a protease-sensitive dipeptide linker to monomethyl auristatin E (MMAE). MMAE is a microtubule-disrupting agent resulting in decreased cell proliferation, induced cell cycle arrest and triggered apoptosis [7]. The safety and the high response rates of SGN-35 as a single agent in clinical trials for relapsed/refractory HL and ALCL, which have been demonstrated in recent studies, led to the quick approval by the Food and Drug Administration to treat lymphoma patients [8]. Since then, it has been widely used for CD30+ lymphoma therapy and has been shown that it can be reused in clinical applications [9]. Another approved method to treat cancer cells is by the use of specific natural killer-like T cells, which are called cytokine-induced killer (CIK) cells. The protocol for generating these cells was created by Schmidt-Wolf et al. in 1991 [10]. After maturation, the heterogeneous cell population consists to a high percentage of CD3^+^CD56^+^ cells but also in some smaller amounts of CD3^+^CD56^−^ and CD3^−^CD56^+^ cells [11]. Among the subsets of CIK cells, CD3^+^CD56^+^ cells have the highest cytolytic capacity, being mediated by a major histocompatibility complex (MHC)-unrestricted mechanism [12]. The exact mechanism of how CIK cells kill tumor cells remains unclear. Studies have demonstrated that CIK cells possess highly cytotoxic activity against numerous tumor cells in vivo and in vitro. Additionally, it has been shown that they can be expanded rapidly [7,13,14,15]. The aim of this study was to increase the efficacy of CIK cells and SGN-35 towards various cell lines when used in combination.

## 2. Results

### 2.1. Effect of CIK Cells on Lymphoma Cells

**Figure 1:**
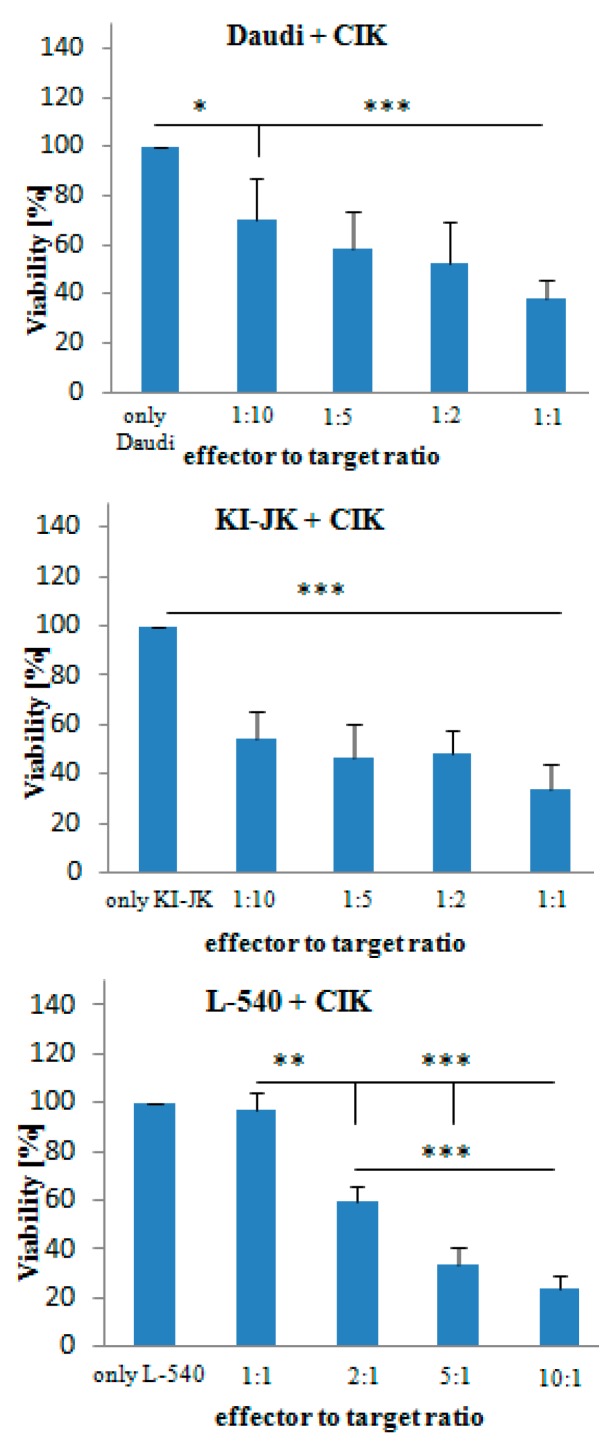
Cytotoxic effect of CIK cells on lymphoma cell lines *Daudi*, *KI-JK* and *L-540*. The cells were cultured at various effector:target ratios of 1:10, 1:5, 1:2 and 1:1 (*Daudi.* and *KI-JK*), and 1:1, 2:1, 5:1 and 10:1 (*L-540*) for 24 h. Subsequently, cell viability was measured using an MTT assay. Results represent data from three different buffy coats. For each probe triplicates were used. Data are presented as mean ± SD (*p* < 0.05). One asterisk indicates a *p*-value of 0.04 to 0.05, two asterisks indicate a *p-*value of 0.02 to 0.03 and three asterisks a *p-*value of <0.02.

*Daudi*, *KI-JK* and *L-540* cells were cultured with CIK cells at various effector-to-target ratios. For *Daudi* and *KI-JK*, 1:10, 1:5, 1:2 and 1:1 ratios were chosen, while, for *L-540*, 1:1, 2:1, 5:1 and 10:1. Lymphoma cell lines and the CIK cells were incubated for 24 h (Figure 1). For these experiments, CIK cells of three different buffy coats were used and cultured in triplicates each time. All cell lines showed a significant decrease in the viability of the cells in vitro according to the different ratios.

### 2.2. Effect of SGN-35 on Lymphoma Cells

**Figure 2:**
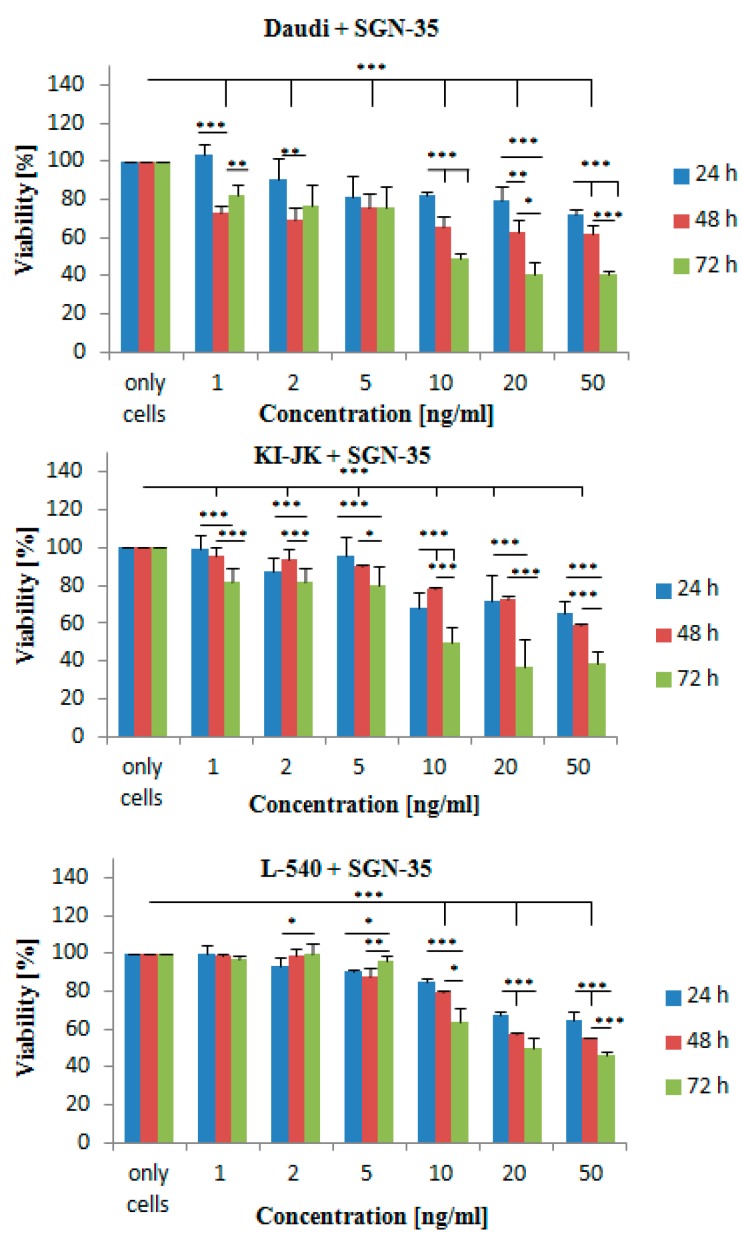
Titration curve of SGN-35 on the different lymphoma cell lines *Daudi*, *KI-JK* and *L-540*. The cell lines were incubated with various concentrations of SGN-35 (1, 2, 5, 10, 20, and 50 ng·mL^−1^) in triplicates for 24, 48 and 72 h. Cell viability was measured using an MTT assay. Data are presented as mean ± SD (*p* < 0.05). One asterisk indicates a *p-*value of 0.04 to 0.05, two asterisks indicate a *p-*value of 0.02 to 0.03 and three asterisks a *p-*value of <0.02.

Lymphoma cell lines were co-cultured with increasing concentrations of SGN-35 (1, 2, 5, 10 and 50 ng/mL) for 24, 48 and 72 h (Figure 2). After 24 h, there was a significant effect on the viability of the cells for the cell lines *Daudi* and *KI-JK* at concentrations >2 ng/mL. For the cell line *L-540*, a significant decrease could be detected at concentrations of >10 ng/mL. IC_50_ ≈ 10 ng/mL were achieved after 72 h. Significance could be seen from concentrations >10 ng/mL. SGN-35 did not have an effect on the viability of the CD30^−^ control cell line *OCI-Ly8 LAM53* (data not shown).

### 2.3. Effect of SGN-35 on the Cytotoxicity of the CIK Cells towards Lymphoma Cells

**Figure 3:**
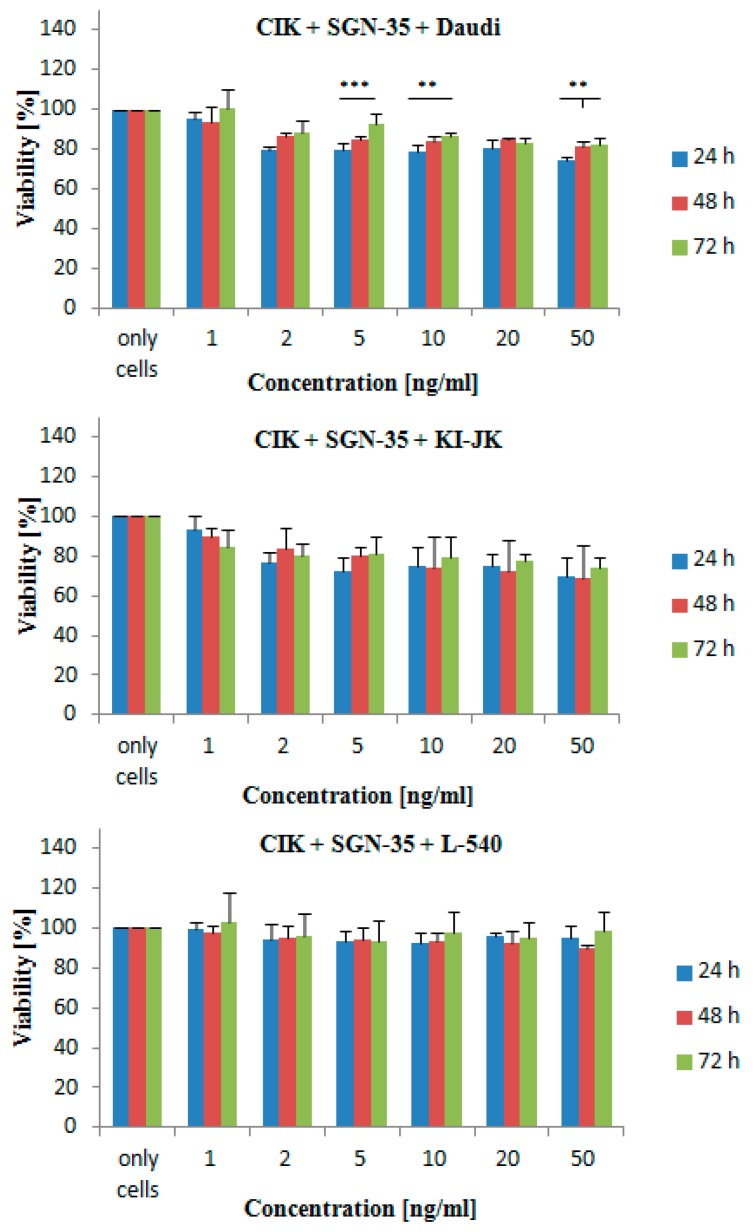
Effect of SGN-35 on the cytotoxicity of the CIK cells after 24, 48 and 72 h. The cytotoxic effect of the CIK cells was tested on the cell lines *Daudi*, *KI-JK* and *L-540* at a 1:1 ratio. The cell viability was measured using an MTT assay. Results represent data from three separate experiments with three triplicates for each probe. Data are presented as mean ± SD (*p* < 0.05). One asterisk indicates a *p-*value of 0.04 to 0.05, two asterisks indicate a *p-*value of 0.02 to 0.03 and three asterisks a *p-*value of <0.02.

To exclude a negative effect of SGN-35 on CIK cell function, various concentrations of SGN-35 (1, 2, 5, 10 and 50 ng/mL) were added to CIK cells. CIK cells from three different buffy coats were used and incubated in triplicates. After 24, 48 and 72 h, the lymphoma cell lines *Daudi, KI-JK* and *L-540* were added and the viability was tested in vitro using an MTT assay. The results show that SGN-35 has no significant effect on the cytotoxicity of CIK cells towards the different lymphoma cell lines, except for *Daudi* (Figure 3).

### 2.4. Combination Experiments of SGN-35 with CIK Cells

**Figure 4:**
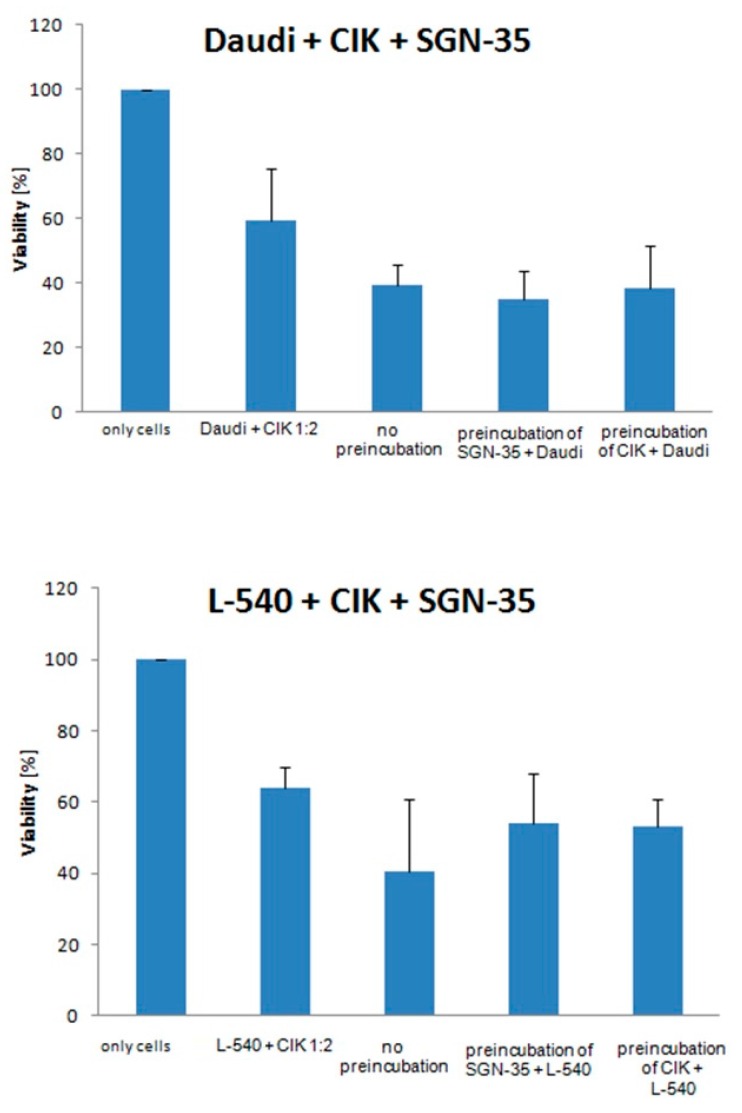
The effect of a suboptimal number of CIK cells (1:2 for Daudi and KI-JK and 2:1 for L-540) and a suboptimal concentration of SGN-35 (10 ng·mL^−1^) on the cell lines. The cell lines were once preincubated with CIK cells only and once with SGN-35 only. After 24 h, the SGN-35 and the CIK cells, respectively, were added and incubated for 72 h. In another experiment, the lymphoma cell lines were incubated with CIK cells and SGN-35 for 72 h without preincubation. As a control, the lymphoma cells were also incubated with CIK cells only. The results represent data from three different buffy coats and were done in triplicates each time. Cell viability was measured with an MTT assay. Data are presented as mean ± SD (*p* < 0.05).

To evaluate additive or synergistic effects of SGN-35 and CIK cells on lymphoma cell lines, a suboptimal number of CIK cells and a suboptimal concentration of SGN-35 were cultured with lymphoma cell lines. For the suboptimal number of CIK cells ratios, 1:2 (*Daudi* and *KI-JK*), and 2:1 (*L-540*), were chosen as well as 10 ng/mL of SGN-35. Three separate experiments were conducted with three triplicates each. Once, lymphoma cell lines were preincubated with SGN-35 and next lymphoma cell lines were preincubated with CIK cells for 24 h before being adding together. In the third experiment, no preincubation was done and lymphoma cell lines, SGN-35 and CIK cells were added simultaneously. Each experiment was conducted with CIK cells from three different buffy coats. Seventy-two hours after the lymphoma cell lines, SGN-35 and CIK cells were added together and an MTT assay conducted. The results in Figure 4 show that the viability of *Daudi* when cultured without any preincubation was approximately 66%, when preincubated with SGN-35 59% and when preincubated with CIK cells 64%. In all three experiments, a significant decrease in the number of lymphoma cell lines could be observed. For the cell line *KI-JK*, only for the preincubation with SGN-35 a significant difference could be seen. The results of the cell line *L-540* showed the best result for the pre-incubation with CIK cells, which resulted in a significant decrease to 63%. The results of the combinational treatment with all three cell lines showed an additive effect concerning the effect on vitality of lymphoma cells.

## 3. Materials and Methods

### 3.1. Cell Lines and Culture Conditions

Three different CD30^+^ lymphoma cell lines (*Daudi*, *KI-JK*, and *L-540*) and as a control the CD30^−^ B cell lymphoma cell line *OCI-Ly8 LAM53* were used (all obtained from Deutsche Sammlung von Mikroorganismen und Zellkulturen GmbH (DSMZ) Braunschweig, Germany). All cell lines were cultured in RPMI-1640 medium (PAN Biotech, Aidenbach, Germany) with 1% penicillin/streptomycin (P/S) (Life Technologies, Darmstadt, Germany). The medium of *Daudi* and *OCI-Ly8 LAM53* contained 10% heat inactivated (hi) fetal calf serum (FCS) (Life Technologies), whereas the medium of *KI-JK* and *L-540* contained 20% FCS (Life Technologies). The cells were incubated at 37 °C and 5% CO_2_.

### 3.2. Generation of CIK Cells

Cytokine induced killer cells were generated in vitro from human PBMC according to the standard protocol developed by Schmidt-Wolf et al. in 1991 [6]. In short, non-adherent Ficoll-separated (Lymphoprep, PAA) human PBMC were cultured in RPMI-1640 medium containing 10% heat-inactivated FCS, 25 mmol/L Hepes (PAA), 1% P/S. Next, (5 × 10^6^) cells/mL were seeded out. On Day 0, 1000 U·mL^−1^ interferon gammy (IFN-γ) (ImmunoTools, Friesoythe, Germany) was added to generate CIK cells. Then, 300 U/mL interleukin-2 (IL-2), 100 U/mL interleukin-1β (IL-1β) (both ImmunoTools) and 50 ng/mL anti-CD3 (α-CD3) (eBioscience, Frankfurt, Germany) were added after 24 h. Every three days some medium was exchanged and 300 U/mL IL-2 was added again. After two weeks CIK cells were mature and ready to use. The cells were incubated at 37 °C in humidified 5% CO_2_ atmosphere.

### 3.3. Antibody–Drug Conjugate

The antibody–drug conjugate brentuximab vedotin (SGN-35), which was kindly obtained from Millennium Pharmaceuticals (Cambridge, MA, USA), was used in this study. The antibody–drug conjugate had a concentration of 4.8 mg/mL from which various concentrations were prepared with the RPMI-1640 culture medium of the CIK cells. One microliter was added to the cells into the 96-well plates and the cells were treated with these concentrations for 24 to 72 h.

### 3.4. MTT Assay

An MTT assay is a quick and easy method to quantitate the number of viable cells. This assay makes use of the ability of viable cells to convert 3-4,5 dimethylthiazol-2,5 diphenyl tetrazolium bromide, which is a soluble tetrazolium salt, into an insoluble formazan precipitate [10].

After counting the lymphoma cells, 50 µL containing 1.5–45 × 10^4^ cells were seeded out in round bottomed 96-well plates in triplicates and co-incubated with various concentrations of SGN-35 and CIK cells over 24 to 72 h before the MTT reagent was added. After 45 to 90 min of incubation, plates were centrifuged for 8 min and the supernatant was discarded. Eighty microliters of DMSO (Carl Roth GmbH & Co. KG, Karlsruhe, Germany) was added. The plate was then centrifuged at 350 rpm for 10 min until the colorimetric analysis could be conducted on a multiwall scanning spectrophotometer (FluoAsterisk Optima, BMG Labtech, Ortenberg, Germany) at 560 nm.

### 3.5. Statistics

Microsoft Office Excel 2007 was used for statistical analysis. To analyze statistical significance, a one-way analysis of variance (ANOVA) was performed. *p-*Values <0.05 were considered as significant. One asterisk indicates a *p*-value of 0.04 to 0.05, two asterisks indicate a *p*-value of 0.02 to 0.03 and three asterisks a *p*-value of <0.02.

## 4. Discussion

Application of CIK cells as well as the use of the antibody–drug conjugate SGN-35 are single alternative treatments for lymphomas that are already approved and have shown clinical efficacy. For SGN-35, a clinical trial showed that 75% of the patients showed partial or complete remission. For the application of CIK cells, a trial in 2014 showed that 50% of the patients changed from partial to complete remission after the infusion of CIK cells. Thus, both methods are good alternative treatments, especially for patients who have refractory disease after chemotherapy, who are not candidates for autologous stem cell transplantation (ASCT), or who relapse after ASCT [12,13,16]. In vitro experiments of previous studies showed that even a really low effector to target ratio of CIK cells and the *Daudi* cell line resulted in a significant decrease of the viability [9]. For KI-JK and L-540, no studies on the cytotoxic effect have been conducted so far. For SGN-35, it has been shown that a concentration of <10 ng/mL is sufficient for decreasing the viability of tumor cell lines to 50% [4].

This study aimed at increasing the anti-lymphoma effect of CIK cells in combination with SGN-35 on three different CD30^+^ lymphoma cell lines (*Daudi*, *KI-JK* and *L-540*) in vitro. Our study confirmed that the viability of lymphoma cell lines can be decreased by CIK cells significantly. Even very low amounts of CIK cells such as an effector to target ratio of 1:10 showed a decrease in the viability of the tumor cell lines *Daudi* and *KI-JK* showing that CIK cells are highly effective against these lymphoma cell lines. The cell line *L-540* was more resistant showing a significant decrease at an effector to target ratio of 2:1 (Figure 1). Tumor cell lines are mainly lysed by the CD3^+^CD56^+^ subset of CIK cells due to a MHC-unrestricted mechanism [8]. The difference in the efficiency towards the different CD30^+^ cell lines might be due to the various anti-tumor mechanisms, such as effector to target-cell contact, signaling and tumor apoptosis via the Fas ligand of CIK cells, which might be not as effective for each cell line [6,9,14,15]. To understand the elimination of tumor cells due to CIK cells completely, the complex mechanism has to be further analyzed.

Furthermore, the literature value of IC_50_ < 10 ng/mL for SGN-35 was confirmed. After 72 h of incubating the lymphoma cells with SGN-35, the results are in accordance with the literature [4]. The negative control cell line *OCI-Ly8 LAM53* was not affected by SGN-35. The results in this study show that at a concentration of 10 ng/mL, 49% and 63% (*L-540*) of the lymphoma cell lines were alive. We did not check on the CD30 expression of our lymphoma cells. However, the possibility of the amount of CD30 surface antigens playing a role in the sensitivity of SGN-35 against the tumor cells has already been excluded by a fluorescence-activated cell sorter analysis in previous studies conducted by Francisco et al. in 2003. For shorter incubation times, the viability of the tumor cells was higher. The reason for that might be the working mechanism of the MMAE that is attached to the mAb molecule. The MMAE needs to be internalized by endocytosis, subsequently released inside the cell so that it can act on the microtubule network and induce cell cycle arrest and apoptosis [17]. Factors like the rate of internalization, enzymatic cleavage, release of MMAE, etc. may play a role on the sensitivity of the cell lines towards the antibody–drug conjugate. In summary, the complete mechanisms of SGN-35 remain unclear. Tumor cell killing via antibody dependent cellular phagocytosis (ADCP), direct effects on tumor cell signaling as well as the diffusion of free MMAE out of the targeted CD30^+^ tumor cells may play an important role in the function of the antitumor activity of SGN-35 [18].

To exclude a negative effect of SGN-35 on CIK cells, the experiments that led to the results in Figure 3 were conducted. No significant effect of SGN-35 on the function of CIK cells could be detected. Nevertheless, a small decrease in the cytotoxicity was obtained. Only for the *Daudi* cell line a significant decrease could be observed for 5, 10 and 50 ng/mL. Thus, the viability of cells was only decreased by a maximum of 8%. This might have different reasons. On the one hand, CIK cells might be highly sensitive to the free particles of MMAE like many other cells [4]. Furthermore, the different mechanisms of the antitumor activity of CIK cells like signaling might be intervened by SGN-35 resulting in a decreased function towards cell lines depending mostly on that mechanism [9]. Even though the SGN-35 did mildly affect the activity of the CIK cells after some time, the results of the previous study could be confirmed stating that SGN-35 is by far not as active against antigen-negative cells as it is against CD30^+^ cell lines [4].

Finally, the anti-lymphoma effect of SGN-35 and CIK cells in combination was investigated in this study. Both methods are already approved treatments to fight lymphomas in vivo and are quite effective [5,9]. In this study, it was aimed to increase the efficacy by combining both methods. The results show a significant additive effect on the viability of the CD30^+^ lymphoma cell lines in vitro in an MTT assay (Figure 4). Cell viability was significantly decreased of the lymphoma cell line *Daudi*, whereas the experiment with the preincubation of the cell line with SGN-35 gave the highest decrease. For the cell line *KI-JK*, a significant decrease could be only observed when the tumor cells were preincubated with SGN-35. For L-540, the same was the case for the experiment with the preincubation with CIK cells. These results indicate that the anti-lymphoma effect towards the different cell lines was significantly higher when used in combination. The previous experiments showed that SGN-35 was not as effective towards the *L-540* cell line as it was towards *Daudi* and *KI-JK*. This might be an explanation for the different results of the combinational treatment towards *L-540.* A reason might be that the rate of internalization of *L-540* is lower than the one of the other cell lines. Since the cell lines were only preincubated with SGN-35 for 24 h, the time frame might be too short for the lymphoma cells of *L-540*. All three cell lines showed that a preincubation of either SGN-35 or CIK cells with the tumor cells is beneficial. A reason for the better results with the preincubation of SGN-35 might be that the SGN-35 induces cell cycle arrest of lymphoma cells, which the CIK cells, when added, can help to lyse and improve the results [6]. Preincubation of CIK cells did mainly lead to the lysis of lymphoma cells without inhibiting their growth by intervening with their cell cycle [9]. The additive effect of the combinational treatment has been shown in this study. To get the best results, further investigations on the time point of combining both methods, as well as the exact working mechanisms of both methods must be conducted.

In summary, the combination of SGN-35 and CIK cells seems to be a promising alternative method to treat lymphomas that are CD30^+^. In the future, the perfect timing for the application of CIK cells and SGN-35 due to their complex working mechanism should be investigated. Furthermore, the exact mechanism and efficacy of this possible alternative method needs to be analyzed in an in vivo model. Another interesting approach would be to combine SGN-35 with the PD1 antibody nivolumab, which is also approved for lymphoma.
